# A Blockchain Framework for Scalable, High-Density IoT Networks of the Future

**DOI:** 10.3390/s25092886

**Published:** 2025-05-03

**Authors:** Alexandru A. Maftei, Adrian I. Petrariu, Valentin Popa, Alexandru Lavric

**Affiliations:** Computers, Electronics and Automation Department, Stefan cel Mare University of Suceava, 720229 Suceava, Romania

**Keywords:** blockchain, Hyperledger Besu, Internet of Things (IoT), massive data storage, scalability, security, QBFT

## Abstract

The Internet of Things has transformed industries, cities, and homes through a vast network of interconnected devices. As the IoT expands, the number of devices is projected to reach tens of billions, generating massive amounts of data. This growth presents significant data storage, management, and security challenges, especially in large-scale deployments such as smart cities and industrial operations. Traditional centralized solutions struggle to handle the high data volume and heterogeneity of IoT data, while ensuring real-time processing and interoperability. This paper presents the design, development, and evaluation of a blockchain framework tailored for the secure storage and management of data generated by IoT devices. Our framework introduces efficient methods for managing, transmitting, and securing data packets within a blockchain-enabled IoT network. The proposed framework uses a gateway node to aggregate multiple data packets into single transactions, increasing throughput, optimizing network bandwidth, reducing latency, simplifying data retrieval, and improving scalability. The results obtained from rigorous analysis and testing of the evaluated scenarios show that the proposed blockchain framework achieves a high level of performance, scalability, and efficiency while ensuring robust security being able to integrate a large number of IoT devices in a flexible manner.

## 1. Introduction

In recent years, the Internet of Things (IoT) has revolutionized the way we interact with our environment, creating a vast network [1] of interconnected devices that are reshaping industries [2], cities [3], and homes [4]. From smart thermostats and medical devices monitoring people’s health [5] to industrial sensors [6] and autonomous vehicles [7], IoT devices have been introduced into every aspect of modern life. This network of IoT devices has vastly expanded leaving behind simple individual use and evolving into global systems and networks with millions of such devices. If this process of expansion continues, it is estimated that the number of active IoT devices will reach tens of billions [8] in the years to come, with commercial deployments used by the everyday user to highly complex industrial system deployments.

The use of IoT devices is not just limited to their deployment in everyday applications used by every person. The number of large-scale deployments of these devices in industrial operations such as agriculture [9], transportation [10], and urban infrastructure [11] is growing. In this context, IoT data servers are the backbone of emerging smart cities, where IoT sensors and devices are being used to monitor and manage everything from traffic congestion [12] and pollution [13] to energy consumption [14] and public safety [15]. This rapid growth and adoption of IoT devices across different areas can lead to improved efficiency, sustainability, and quality of life. However, large numbers of devices in the order of tens and hundreds of thousands or even millions of devices can introduce some significant challenges such as data storage [16], management [17], and security [18].

Usually, the IoT ecosystem is associated with a massive and ever-growing volume of data. In most cases, IoT devices collect and transmit data in a continuous manner, thus providing real-time insights that can help the process of decision making and optimization [19]. However, an exponential increase in active IoT devices leads to a similar increase in the amount of data they produce that needs to be stored and sometimes archived for further references. With hundreds of thousands or millions of IoT devices operating in large urban areas or industrial environments, the amount of data generated can become quite overwhelming for current storage systems.

This massive volume of IoT data presents some major challenges related to how to store, manage, and analyze it in the most efficient and secure way. Traditional storage and management solutions are often not well suited to satisfy the requirements of these massive, large-scale IoT networks and systems. The massive volume of IoT data generated by these devices, combined with the need for real-time processing, requires a change in the current approach of data storage and data security.

In addition, data from IoT devices are not homogeneous, as they have different packet formats and come from multiple sources such as sensors, cameras, and wearable devices. Most of the time, these devices operate in an independent manner without user interaction and each collects data that are sent and isolated within its own ecosystem. This makes it difficult to integrate data from multiple IoT devices developed by different vendors into a unified system or network, complicating efforts to solve storage and analysis problems. Additionally, IoT devices often operate using various standards and communication protocols [20], making it challenging to ensure seamless interoperability between different systems. The constant flow of data puts a lot of pressure on the IoT network infrastructure that manages the devices and data packets. As the number of active IoT devices grows by the day, they require a robust and scalable network that can handle the transmission of a large volume of data in real time. This need for continuous and reliable connectivity further complicates efforts to efficiently manage data packets.

In this paper, we propose a scalable, blockchain-based framework designed to securely store, manage, and retrieve data generated by large-scale IoT deployments. With the exponential growth of IoT systems in urban, industrial, and environmental contexts, the need for secure, high-throughput, and tamper-resistant data management solutions has become increasingly important. Traditional centralized architectures struggle to accommodate the massive volume and velocity of IoT data while maintaining trust, integrity, and availability. To address these limitations, this work leverages blockchain’s inherent characteristics such as immutability, decentralization, and transparency, to build a robust and future-ready data management layer for IoT ecosystems.

The framework facilitates the structured transmission of data packets from multiple IoT devices to a permissioned blockchain network, where the data are securely stored and indexed. A user-facing dashboard web application complements the backend system, allowing authorized users to visualize, filter, and interact with the data in real time. Efficient data retrieval mechanisms and dynamic gateway behavior ensure the system remains performant under high data loads.

The specific contributions of this work are as follows and are expanded upon in subsequent sections:

- A vertically integrated framework has been developed, that includes IoT sensor layers, gateway nodes, a permissioned blockchain network using QBFT consensus, and a web-based visualization interface. The system is designed to support networks ranging from a few thousand to over one 1,000,000 IoT devices.
- We propose a blockchain gateway design, capable of buffering, sorting, and aggregating data packets based on criteria such as timestamp or device ID. The gateway batches multiple data packets into transactions, significantly reducing transaction overhead and optimizing blockchain performance.
- A hierarchical binary search strategy is integrated at the data retrieval node level to ensure fast access to stored data, even as the blockchain ledger grows. This approach improves the query efficiency to O (log n) over conventional linear searches.
- The proposed framework is experimentally validated using five deployment scenarios ranging from 5000 to 1,000,000 IoT devices. Performance metrics such as IoT data packet throughput, transaction latency, block size, and blockchain ledger size are analyzed to demonstrate scalability and robustness.
- A critical analysis of blockchain ledger size over time is provided, highlighting the impact of IoT data packet volume, block time, and batching strategy.

The rest of the paper is organized as follows: In Section 2, we outline the advantages of applying blockchain technology to IoT systems and networks. Section 3 reviews the current state of the art in various architectures that integrate blockchain technology with IoT systems in different ways. In Section 4, we introduce the design of the proposed novel blockchain IoT framework, focusing on the key components that make up the system. We also describe the primary roles of these components, and the functions implemented within the proposed framework. Section 5 provides a detailed explanation of the implementation of our framework, including the configuration options used for setting up the blockchain IoT system. Section 6 presents the evaluation scenarios and benchmarks, along with the results obtained. This includes metrics such as data packet throughput, latency, and optimal configurations for block and transaction sizes. In Section 7, we discuss the security and scalability aspects of the proposed framework. Finally, Section 8 provides the conclusion of the paper, along with suggestions for future development and implementation.

## 2. Blockchain Advantages in IoT Large-Scale Networks

Blockchain technology is the solution that can solve many of the problems associated with the Internet of Things, specifically the problems related to the storage, management, and security of the IoT. Its unique characteristics such as decentralization, immutability, transparency, distributivity, and high security make it a very good solution to reduce the problems that arise from the growing number of IoT devices.

One of the main advantages of blockchain technology is its decentralized nature. Most of the time, traditional IoT data storage solutions rely on centralized data centers, which can represent bottlenecks and single points of failure. In contrast, blockchain technology distributes data packets across a network of nodes, making them less susceptible to cyberattacks [21] or data manipulation.

Blockchain decentralizes and distributes data packets, reducing the reliance on centralized storage often used by IoT systems. In a blockchain network, each IoT device helps store and share data across the entire system. Instead of relying on one central location to hold the data, the devices work together to store data in a decentralized way, creating a unified and consistent record across the network. This improves interoperability, allowing data from various devices spread over large areas to be easily integrated and analyzed together.

Once data have been stored in the blockchain network, the data cannot be modified or deleted without the consent of at least half of the entire network. This immutability feature described above ensures that the data packets collected by IoT devices are tamper-proof, thus ensuring their integrity. This feature is important in environments such as smart cities where data accuracy is essential for more accurate and effective decision making.

Blockchain technology uses cryptographic methods [22] to secure data packets. Each transaction or data packet stored in the blockchain network is encrypted, ensuring that only authorized entities can access it. Moreover, each data packet is linked to the previous one, thus creating a chain of trust that improves authentication and reduces the risk of data breaches. The distributed nature of blockchain makes it inherently resilient against cyberattacks. Attackers need to take control over most of the nodes of the blockchain network to subsequently modify data packets. However, this is very difficult compared to traditional centralized systems for storing data packets.

Blockchain technology offers innovative solutions to many of the problems that are due to the rapid growth of IoT devices. By decentralizing the storage process, improving the security and integrity of data packets, promoting transparency, and facilitating automated processes, blockchain can significantly improve the process of managing the massive volume of data packets generated by IoT systems and networks.

## 3. Related Works

Numerous studies have explored the integration of blockchain technology into large-scale IoT networks, proposing a range of architectures and mechanisms aimed at enhancing data storage, security, and system reliability. Therefore, in this section we present a structured overview of the most relevant approaches in the recent literature, highlighting their design principles, core contributions, and the specific challenges they address.

A hybrid blockchain–IoT architecture combining Ethereum smart contracts, IPFS for off-chain storage, and attribute-based encryption (ABE) to enforce fine-grained access control was proposed by Ullah et al. [23]. It introduces a decentralized data-sharing model where users can manage, monetize, and securely distribute IoT data. The design integrates smart contracts for permission control and FileCoin for incentivized storage. While the framework offers strong privacy and access guarantees, its reliance on off-chain infrastructure introduces latency and complexity in data retrieval and synchronization. Additionally, the system lacks dedicated mechanisms for efficient indexing or gateway-level optimization, which are essential for high-frequency IoT data flows. Performance was evaluated on the Rinkeby Testnet, but no large-scale simulation or real-time stress testing was conducted.

Na et al. [24] propose a federated blockchain architecture aimed at reducing latency and improving reliability in IoT decentralized applications. Their system clusters blockchain nodes into service units, each managed by a rotating leader node that coordinates local data storage and metadata synchronization. Unlike IPFS-based models, this architecture stores actual files within local nodes in the blockchain network, significantly reducing upload and download latency. Experimental results demonstrate up to a 10× performance improvement over traditional off-chain storage. The use of round-robin leader rotation also mitigates centralization bottlenecks. However, scalability remains a concern, as the architecture depends on node-local storage without dynamic scaling mechanisms or ledger size optimization. The implementation relies on a private Ethereum-compatible network with Proof of Authority (PoA), which may limit decentralization.

In another paper proposed by Basudan et al. [25] presents a layered blockchain architecture tailored for secure transaction handling in dynamic IoT environments. The system is structured across cloud and fog layers and employs certificate-based authentication and Role-Based Access Control (RBAC) to enforce trust and access management. Smart contracts are utilized to control data flow and validate device identities within the network. The framework aims to address device trust, integrity, and the secure exchange of data in IoT ecosystems. Its modular design is suitable for managing heterogeneous devices and dynamic participation, with an emphasis on security and hierarchical processing. The authors also explore the application of smart contracts for data validation and establish a secure chain of communication between IoT devices and blockchain nodes.

A performance-adaptive blockchain model named Light-PerIChain tailored for heterogeneous IoT environments was proposed by Fathi et al. [26]. The key innovation lies in its dynamic classification of nodes based on their computational capabilities, allowing each node to take on roles suited to its resources. The proposed Lightweight Consensus Algorithm (LCA) enhances energy efficiency and ensures minimal load on constrained devices. The paper highlights an adaptive approach to resource distribution, aiming to maintain a balanced and scalable network. It demonstrates the system’s capacity to accommodate resource diversity without degrading performance, with evaluations confirming gains in throughput and reduced computational load. This design is particularly suitable for deployments where nodes vary significantly in power, such as in wearable, edge, and remote sensing applications.

Another paper proposed by Mahmoud et al. [27] presents a modular blockchain model optimized for low-power IoT environments. The architecture is composed of four main modules: a scalable blockchain core, a lightweight consensus algorithm (LCA), changeable cryptographic functions (CCF), and a throughput management system (TMS). Together, these modules aim to support flexible, energy-efficient, and adaptable blockchain deployments, particularly in resource-constrained settings like smart homes or lightweight industrial IoT. The paper highlights the potential of combining configurability and efficiency, allowing different components to be tailored based on application needs. Performance testing using NS-3 simulations demonstrates the framework’s ability to balance energy consumption with operational effectiveness.

Dhulavvagol et al. [28] present a high-performance blockchain framework using hybrid shard generation and static analysis-based data partitioning to improve scalability and network responsiveness. The system is designed to parallelize transaction execution by distributing it across multiple dynamically formed shards, each governed by a tailored consensus mechanism. A core innovation lies in the use of static analysis tools to guide data partitioning, ensuring that transaction dependencies are respected, and cross-shard communication is minimized. The evaluation, based on historical Ethereum data and Hyperledger Caliper, demonstrates significant improvements in transaction throughput and latency, with miners and validators obtaining performance gains approaching twice the baseline. The paper further explores workload balancing, shard creation logic, and protocol-level optimizations for transaction grouping.

The reviewed literature presents a wide spectrum of approaches to integrating blockchain in IoT ecosystems, each addressing specific facets such as access control through attribute-based encryption, energy efficiency in constrained devices, or architectural modularity using edge computing and shard-based designs. However, despite these contributions, a critical gap remains across all works: the lack of a cohesive, scalable solution capable of supporting high-frequency data ingestion, continuous on-chain processing, and efficient data retrieval across large-scale, heterogeneous, high-density IoT networks.

Our proposed framework directly addresses this gap by introducing a vertically integrated architecture optimized for end-to-end performance. Through gateway-level data packet aggregation, dynamic transaction batching, binary search-based retrieval, and a permissioned blockchain with QBFT consensus, the framework enables high-throughput, low-latency processing suitable for environments with tens or hundreds of thousands of concurrently active IoT devices. Unlike the reviewed solutions, which either remain theoretical, rely heavily on off-chain dependencies, or lack scalability validation, our system has been rigorously tested under emulated load conditions, demonstrating both robustness and adaptability.

In summary, while existing works each contribute valuable perspectives to the blockchain-IoT landscape, they often fall short in providing an integrated and performance-validated solution for large-scale deployments. Our work addresses this gap by providing a practical, scalable, and efficient platform explicitly designed for the demanding scenarios of high-density IoT environments.

## 4. Novel Blockchain IoT Framework Design

In this section, we present the proposed framework, which utilizes blockchain technology and its key features to provide a highly secure solution for large-scale IoT systems and networks. By leveraging blockchain’s inherent security, traceability, and immutability, the framework ensures reliable storage and management of the vast number of data packets generated by diverse devices and sensors within massive IoT networks.

**Figure 1:**
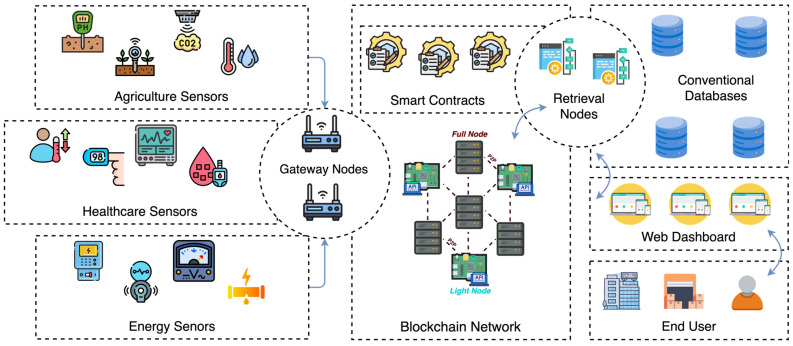
Detailed overview of the proposed blockchain-integrated framework for securing large-scale IoT systems.

Our framework is composed of several essential components, including IoT sensors, gateway nodes, blockchain network, retrieval nodes, bootnode, smart contracts, conventional database, and a dashboard web application. As shown in Figure 1, these components work together to deliver seamless functionality and enhanced security for IoT systems.

### 4.1. Framework Components

#### 4.1.1. IoT Sensors

IoT sensors deal with taking various information from the environment and transmitting it at regular intervals to the gateway. The IoT sensors can use different communication protocols such as Wi-Fi, Sigfox, and LoRa for a wide variety of communication. These protocols enable sensors to transmit data efficiently, depending on factors like range, power consumption, and data rate. Additionally, the choice of protocol cannot influence overall network framework performance, allowing for flexibility in deployment based on specific use cases. By utilizing diverse communication methods, IoT sensors ensure reliable connectivity and adaptability to different environmental conditions and requirements.

Each IoT device has a universally unique identifier (UUID) [29] that is added in the sent data packet and is used for further device identification. The data field from a transaction contains its value and is encoded in hexadecimal format. This field is often used when calling a smart contract, which is also the case for our framework. The value in this field is divided into two components: the function selector and the function parameters.

Function selector: Refers to the first 4 bytes of a transaction and is used to identify the specific function being called within a smart contract. In this context, it corresponds to a function that accepts multiple parameters.

Function parameters: The remaining values following the function selector, representing the input data that are passed to the smart contract function for execution.

#### 4.1.2. Gateway Nodes

Each gateway runs continuously and waits for data packets from IoT devices. The proposed gateway has several integrated functionalities, such as the following: (1) manages the nonce value (a sequential value to order transactions) to sign transactions; (2) ensures that transactions are signed and sent with the correct nonce value to prevent replay attacks; (3) supports communication protocols such as HTTP and WebSocket, making it flexible for different IoT devices; (4) sends transactions in rotation to the blockchain nodes, ensuring that even if a node goes down, other nodes can still receive the data packets.

Gateway nodes collect data packets from IoT devices and store them in a data stack/memory buffer. When the gateway buffer reaches its limit, it takes the data from the stack and sorts them using a sorting algorithm using certain criteria such as device ID and timestamp and then prepares them by encapsulating them in a transaction to be further sent to a node in the blockchain network for permanent data storage. Transactions sent by the gateway nodes are signed by them using their own private key for security and faster identification and search.

When the data have been taken from the stack, the stack will be emptied to be able to add the next data packets that are sent by the IoT sensors. Similar to the interaction between sensors and gateway, the interaction between blockchain network nodes and the gateway can be achieved through communication protocols such as webhook (HTTP request), HTTPS, MQTT, or 5G.

#### 4.1.3. Blockchain Network

The blockchain network in our proposed framework is used to store information. The nodes of the blockchain network have high computational power to communicate as efficiently and seamlessly as possible with the gateway nodes and the data packet retrieval nodes. The blockchain network also checks the integrity of the data packets. The verification performed by the nodes of the blockchain network is done through the consensus mechanism, in this case Quorum Byzantine Fault Tolerance (QBFT) [30].

At the blockchain network level, we have two types of nodes, namely Full Nodes and Light Nodes. Full Nodes deal with storing data, verifying transactions, and maintaining a complete history of the blockchain chain of packet data. These nodes can reach very large sizes in the order of terabytes, the reason being that these nodes contain a complete history of all interactions within the blockchain network and are connected to power mains. Light Nodes, as the name implies, are blockchain nodes that are part of the network but do not have the same processing capabilities as Full Nodes. Because of this, these nodes do not contribute to the validation process of transactions to be stored in the network. They rely on Full Nodes for transactions validation and data query. They can be seen as intermediaries through which to interact with the blockchain network. These nodes are used for cases when an entity wants to connect to the network to query it about data packets sent by IoT devices. In this case, the simplest and least expensive solution in terms of storage is a Light Node because through it, the blockchain network can be easily queried. Moreover, gateways can also connect to these nodes to send data packets encapsulated in transactions. However, one must consider the delays that may occur because such a node sends the transactions for confirmation to the Full Node. When it comes to communication protocols, the Peer-to-Peer (P2P) protocol is the fastest communication protocol that can be used in the blockchain network.

#### 4.1.4. Smart Contract

The Smart Contract (SC) interfaces the connection between the gateway nodes and the blockchain network to enable data storage. It also communicates with nodes that have data retrieval algorithms. To support secure and structured on-chain storage of IoT data, we developed a lightweight and efficient smart contract using the Solidity programming language. The contract is designed to store data packets submitted by gateway nodes, which aggregate sensor readings from a large number of IoT devices. Its structure prioritizes simplicity and fast interaction, while enabling persistent storage and data retrieval. The smart contract defines a *DataPacket* structure that contains two fields: a timestamp indicating when the data were generated, and a *jsonData* array containing one or more entries in serialized JSON format. Each IoT device is uniquely identified by a *sensorId*, which serves as a key in a mapping that links each sensor to an array of its associated data packets. The primary function, *storeData*, accepts a sensorId, a timestamp, and an array of *jsonData* strings. When called by the gateway, it validates the input, stores the data in the blockchain, and emits a *DataStored* event for external tracking. All data are permanently recorded, supporting both immutability and auditability.

For retrieval, the contract provides two additional public view functions. *getDataBySensorId* allows users or retrieval nodes to access the full set of data packets for a specific sensor, while *getPacketCount* returns the number of entries stored under that sensorId. These functions enable seamless interaction with the blockchain network for data visualization or off-chain processing. This smart contract acts as the core interface between the blockchain layer and the data management components of the system. It ensures secure, authenticated, and traceable storage of environmental data while allowing efficient access for analysis and monitoring through the dashboard application.

Following the successful deployment of the smart contract within our blockchain IoT network, it will result in its own address through which the smart contract can be called. Specifically, gateway nodes and retrieval nodes will call the data storage and retrieval functions through the unique address in hexadecimal format. The smart contract is built using the Solidity [31] programming language. To be deployed in our network, the hardhat [32] application is used; this is a specially designed and easy-to-use application to interact with smart contracts.

#### 4.1.5. Retrieval Nodes

The data retrieval nodes are nodes designed to retrieve data from the blockchain network and send them to the database where the link between the sensors and their owners will be made. For this reason, the binary search algorithm [33] is also used because it is the fastest algorithm to search for information in a sorted data structure. Each transaction on the blockchain contains multiple data packets, which are pre-sorted based on attributes such as timestamps or UUID. This ordered structure enables the binary search algorithm to operate with logarithmic efficiency, even as the dataset grows over time. When a retrieval request is received, the node begins by accessing the list of transactions, treating it as a sorted array. Two pointers—low and high—define the initial search range, corresponding to the indices of the first and last transactions.

The algorithm calculates the midpoint and inspects the transaction at that position. If the desired data packet is present, the search concludes successfully. If not, the algorithm determines whether the target is in the lower or upper half of the remaining search space and updates the boundaries accordingly. Each transaction, in turn, contains a set of data packets, also sorted. If the requested packet is within the transaction, a second binary search is recursively applied within this inner dataset. The process continues until the packet is found or the search space becomes invalid. If the data packet is not located, the retrieval node notifies the requesting client. This hierarchical search approach reduces the retrieval time significantly, achieving a time complexity of O(log n), which represents a notable improvement over traditional linear search methods.

In the conventional database will be saved all the information about the IoT devices (UUID, timestamp, data packet itself) and the network users to which this device belongs. Both the retrieval nodes and the gateway nodes will mostly communicate with the blockchain network through remote call procedures (RCPs). In this case, we used the web3js [34] library through the JavaScript programming language.

#### 4.1.6. Bootnode

Another important element of the network is the so-called bootnode [35]. This node allows the other nodes in the network to discover each other. In our implementation, only one bootnode was used. The bootnode plays a crucial role in maintaining network connectivity by providing essential information about active nodes and their addresses. Additionally, it facilitates the initial connection process for new nodes joining the network, ensuring a smooth integration. This centralized approach simplifies node discovery, allowing for efficient communication and data exchange among the limited number of nodes in the network.

#### 4.1.7. Conventional Database

The conventional database stores data collected by retrieval nodes, along with user-related information, and makes it accessible for display in the web application’s dashboard. Although blockchain is good and secure data storage technology, when it comes to data retrieval for displaying data over long periods of time, it is not the most efficient solution. To address this shortcoming, we utilize a traditional database solution as a supplementary storage option for data sent by the retrieval nodes, as traditional databases are more efficient for data retrieval. Additionally, we use the traditional database for user–IoT device mapping. However, the primary focus remains on the blockchain network for data storage. The communication protocols employed for this process can include HTTP/HTTPS, 5G, or MQTT.

#### 4.1.8. Dashboard Web Application and Users

Data visualization and user–device interaction for IoT systems are facilitated through a dashboard web application developed with the NodeJS framework [36]. The application is hosted in a cloud environment, ensuring scalability, accessibility, and seamless performance for users. Multiple applications can be deployed for each user, thereby distributing the computational workload and optimizing performance across the system, rather than relying on a single dashboard application.

**Figure 2:**
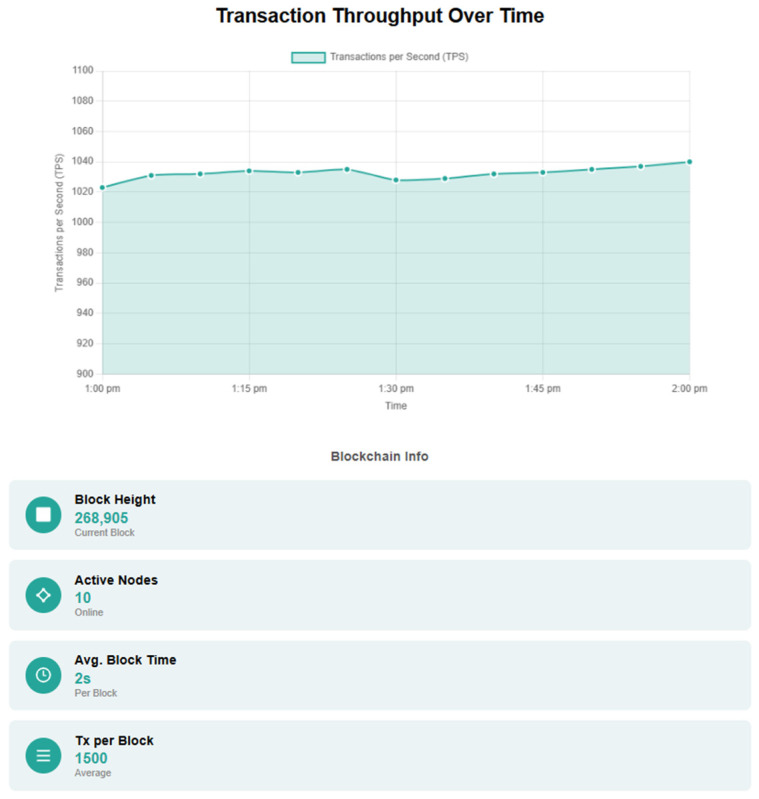
Web-based dashboard application for visualizing IoT data.

The dashboard web application, seen in Figure 2, allows users to visualize the active sensors, to view the data sent by them and to register or enroll new or existing devices within the proposed framework. Moreover, the dashboard web application also provides several metrics that can be visualized, metrics such as total number of data packets sent, total number of transactions, total number of blocks, size of block sent, throughput, transaction latency, and latency of data packets sent.

Furthermore, the dashboard web application offers users a range of advanced search capabilities, including device-specific queries and time-based filters, enabling quick and efficient access to relevant IoT data. These features allow users to easily navigate and analyze their device data with precision.

Users can interact with the blockchain network through the dashboard, where they can visualize information about the sensors integrated into the network and the data transmitted by them. The dashboard provides real-time updates, allowing users to monitor sensor performance and data integrity effectively. Additionally, users can access historical data trends, enabling them to analyze patterns and make informed decisions based on the collected information. Advanced filtering options help users customize their view, focusing on specific sensors or time periods for deeper insights.

### 4.2. Framework Data Flow

**Figure 3:**
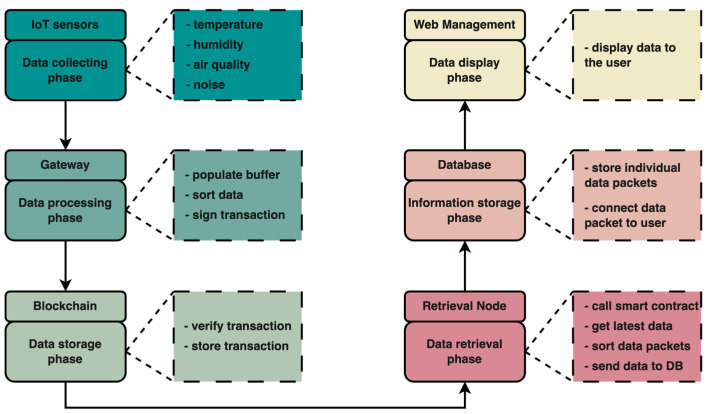
Diagram flow for the data packets gathered by the IoT devices from the environment.

The data flow diagram is illustrated in Figure 3. As shown, each data packet moves through multiple stages, collectively referred to as the packet data flow. The key stages a data packet must pass through include the following:

1. *Data collection phase:* At this level, various sensors collect information from the surrounding environment such as temperature, humidity, air quality, CO2 levels, gas detection, and many others. The sensors are set to send a data packet to the gateway nodes at different time intervals, thus reducing network congestion and reducing stress on the nodes within the network. Again, IoT devices use cryptographic algorithms to encrypt the data packets thus providing data security, as mentioned in Section 2.
2. *Data transmission phase:* The next step is to send the data to gateway nodes. This is performed by means of a communication protocol, and in this case HTTP, HTTPS, and MQTT protocols were used. The use of multiple communication protocols demonstrates the effectiveness of the gateway nodes and the lack of limitation of the proposed framework to a single communication protocol.
3. *Data processing phase:* When the data packets arrive at the gateway nodes, they will be stored in a data stack. Before being forwarded further to the blockchain network for storage, the data packets are sorted, encapsulated in a transaction, and then signed by the gateway node.
4. *Data transmission to the blockchain phase:* After encapsulating the data packets in a transaction and signing it by the gateway node, the transaction is sent to the blockchain network for validation and permanent storage. Specifically, the gateway node will send the transaction to the smart contract, and then it will be added to the blockchain network.
5. *Blockchain storage phase:* The QBFT consensus mechanism is used to validate and store the data in the blockchain network. Although the data are transmitted in a JSON-like structure, the smart contract can accept largely any kind of data structure. Transactions can be sent directly to a Full Node or a Light Node.
6. *Data retrieval phase:* The next step is to retrieve data from the blockchain network. Data retrieval nodes are also used for this process. Performance-efficient search algorithms are implemented at the node level. One such algorithm is the “binary search” which is very efficient if the data packets in a transaction are already sorted, which is already happening at the gateway nodes before the data are transmitted to the blockchain network. Afterwards, the data will be sent to the database to be processed and interpreted.
7. *Data display phase:* Data retrieved from the blockchain network, passed through the search algorithm and included in the database, can be displayed in the web dashboard application to be visualized by each individual user.

## 5. Framework Implementation and Configuration

In this section, we present the detailed implementation steps of our proposed framework. Each subsection addresses a key component of the system, highlighting its role and functionality.

### 5.1. Blockchain Network Implementation

For the implementation of the blockchain IoT network, the Hyperledger Besu v24.7.1 [37,38] client was used. Hyperledger Besu is a client application that uses Ethereum blockchain technology and can be used to develop applications where high performance and high security of transactions within a private network are required. This client features several popular consensus mechanisms such as Proof of Stake, Proof of Work, and Proof of Authority (IBFT 2.0, QBFT, and Clique which are variants of the PoA mechanism). In addition to the wide range of publicly known consensus mechanisms, HB also utilizes the Ethereum Virtual Machine (EVM) [39]. EVM enables the launch and use of smart contracts in a blockchain network.

HB was installed on five virtual machines in a cloud system where each virtual machine runs 2 Hyperledger Besu clients, thus in total 10 clients that act as validators forming the private blockchain IoT network. Each virtual machine in the cloud has an Intel Xeon Processor (Icelake) with six cores, six threads, 32 GB RAM, and 4 TB of storage capacity. The next step was to address some of the blockchain network’s limitations by modifying the genesis file [40] enabling it to better support the IoT scenarios we developed. The first thing was to set the gas limit of the blockchain to a value of 10 billion Wei, which means that in theory, the total limit of the volume of data packets accepted is unlimited. Gas refers to the unit that measures the computational effort required to perform certain operations such as adding data packets from IoT devices to the blockchain network.

However, in practice this is not possible as there might be blockchain distribution problems in the distributed network and high latency. In a blockchain network designed and built for the cryptocurrency domain, removing this limit is not a good solution. However, the reason for raising that gas limitation for blockchain is that we want to use the blockchain network within the IoT system where the number of IoT devices sending data packets can be several million, which leads to a transaction count of several thousand per second. Another setting is where we have chosen the consensus mechanism. In our framework, we have chosen the QBFT consensus mechanism, a variation of the IBFT 2.0 mechanism, with an important parameter such as block time which is the interval at which a new block is produced in the network.

One important aspect to address is the common trade-off between faster block generation and the security of the blockchain network. In public blockchains such as Bitcoin or Ethereum (before The Merge), which rely on Proof of Work (PoW), reducing block time can increase the risk of forks and double-spending, as the network may not reach consensus quickly enough. However, because our proposed framework is built on a permissioned blockchain using the QBFT consensus protocol, these risks are eliminated by design. QBFT does not rely on computational difficulty or mining. Instead, it uses a voting-based mechanism where blocks are added only when at least two-thirds of authorized validator nodes reach consensus. In our system, these validators run on 10 virtual machines in a cloud environment, ensuring secure and authenticated participation.

Because QBFT does not involve mining competition, it tolerates much shorter block times, such as the 2 s interval used in our framework, without sacrificing network stability or data integrity. Furthermore, unless more than one-third of the validators are compromised, QBFT prevents forks and unauthorized data modification. This makes low block time not only safe but beneficial, improving data throughput and responsiveness in high-density IoT environments without compromising security.

Among the consensus mechanisms supported by Hyperledger Besu, such as IBFT 2.0, Clique (PoA), and PoW, QBFT was selected because it provides the best balance between fault tolerance, efficiency, and performance for our specific use case. Compared to Clique, which only tolerates crash faults and assumes honest behavior, QBFT is Byzantine fault-tolerant, making it more robust in the presence of potentially faulty or malicious blockchain nodes. While IBFT 2.0 also provides Byzantine fault tolerance, QBFT improves upon it with enhanced message handling and lower latency, which is particularly advantageous in time-sensitive IoT applications. PoW, on the other hand, was unsuitable due to its high energy consumption and computational demands, which conflict with the lightweight and energy-efficient nature of most IoT deployments. Therefore, QBFT was the most appropriate choice, enabling fast, secure, and resource-efficient consensus in a permissioned environment tailored to the needs of scalable high-density IoT networks.

### 5.2. Nodes Configuration

Other settings that were needed to improve the blockchain network, more specifically the Hyperledger Besu client, were the configuration settings of the 10 nodes that make up the framework we proposed. These settings can be seen in Table 1. The options presented above are configurations that are applied at the level of each node, unlike the configurations made in the genesis file that are applied at the entire level of the blockchain IoT network.

Within the list of individual node configurations, only five of them have a significant impact on improving the performance of the blockchain IoT network. The first setting is x-pool-limit-by-account-percentage which refers to the maximum percentage of the transaction pool that can be utilized by a single account (in this case by a single gateway that transmits data packets to the blockchain network). In the case of our network, for this option, we chose a value of 0.5 which means that a gateway can occupy only 50% of the transaction pool, thus preventing monopolization. The next option is tx-pool which refers to the way transactions are handled in the transaction pool. For this option, we chose the value sequenced, meaning that transactions are processed in a FIFO (first-in-first-out) manner, i.e., in the order in which they are added to the transaction pool. The next option is cache-last-blocks with a value of 32. This option refers to the number of recent blocks that each of the 10 nodes keeps in memory for much faster access. By keeping 32 blocks in memory, a node can respond much faster to different requests from the other elements of the blockchain IoT network. By setting the tx-pool-max-size option to a value of 8192, we have increased the number of transactions that the transaction pool can store. The last modified option is data-storage-format and has been set to a value of BONSAI [41]. This option specifies the storage format used by each individual node for blockchain data. The BONSAI option is an efficient data structure designed to reduce storage requirements and increase data reading performance.

All these options presented in detail have been chosen to provide the highest performance for the framework we propose. The other settings in Table 1 also play an important role, but this time in the communication framework. These settings are applied at the network level to establish the most efficient and trouble-free communication.

### 5.3. IoT Devices Emulation

To test the proposed framework, we emulated a dynamic number of IoT devices using the goroutine principle [42] specific to the Golang programming language. Goroutines are lightweight and efficient threads that allow performing processes in a concurrent manner. This work allowed us to emulate individual IoT devices that transmit single data packets with different parameters such as temperature, humidity, noise level, brightness, and many others. To emulate IoT devices as close to the real world as possible, at runtime, IoT devices choose an interval at which to send data packets; for example, a given sensor may send information to the gateway once every 5 min with a total of 12 data packets sent in an hour interval.

In the proposed framework, IoT devices are arranged in a star topology, where they are connected to a gateway node. The interaction between the IoT devices and the gateway nodes can be done through communication protocols such as 5G, LoRa, Sigfox, MQTT, CoAP, and HTTP/HTTPS. IoT sensors can be implemented by both live and emulation methods; we use the latter method. Both real and emulated nodes can use different communication protocols such as MQTT, LoRa, HTTP, and WebSocket to transmit data packets to our network. Although the communication protocol is not a limitation for our framework, we decided to use HTTP and WebSocket protocol for node communication with the gateway. The emulated IoT devices are configured to transmit information such as UUID, timestamp, and information such as temperature, noise, and oxygen level in the environment. It is necessary for the IoT device to transmit its UUID as part of any data packet as it is used to link the device to its owner. In the dashboard web application, each user can visualize the data from IoT devices integrated in the proposed network. The timestamp is also important; it is in Unix format and is used for the process of sorting and searching data packets. Due to the search algorithm in the retrieval nodes, it is necessary that the data sent to the blockchain should be sorted first; otherwise, the search and finding of the data packets will not be performed correctly.

## 6. Framework Benchmarking and Performance Evaluation

**Figure 4:**
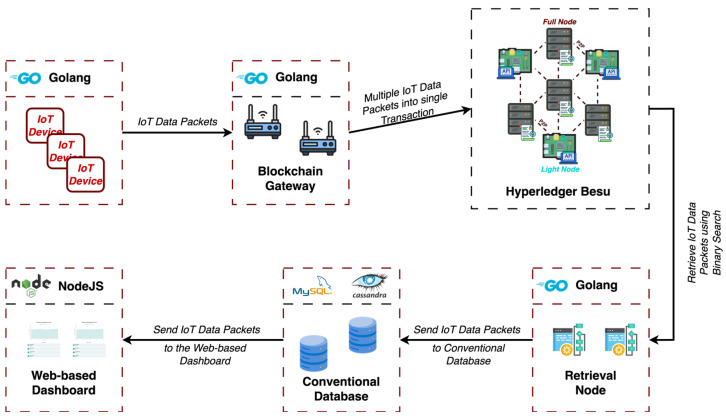
Blockchain-enabled IoT testbed framework used during evaluation.

In this section, we present the steps taken to test the proposed blockchain IoT framework. The main goal following the testing of our proposed framework is to evaluate the practicality and performance of the proposed blockchain network in massive IoT data storage systems. As we specified in the previous chapter, the blockchain network consists of a total of 10 blockchain nodes hosted on five virtual machines within a cloud platform. Figure 4 illustrates the framework of the blockchain IoT testbed used during the evaluation phase. The diagram outlines the interaction between heterogeneous IoT sensors, blockchain gateways, full and light blockchain nodes, and web-based dashboard.

To test the performance and applicability of the blockchain network, in our case Hyperledger Besu, we have evaluated it through five scenarios as close to reality as possible. In addition to the five scenarios, we also analyzed security and scalability, two important characteristics of a blockchain network. In Table 2, we can see the scenarios established to analyze the performance of the blockchain IoT network.

### 6.1. Performance Evaluation Scenarios

The reason for using scenarios (five scenarios in this case) for testing our framework is that we want to show the applicability and importance of using blockchain technology in massive IoT systems, and not only for storing data providing a high level of security. For all five scenarios where the number of emulated devices and the block time were modified, the gateway buffer was also adapted accordingly. In the first phase, we set the gateway buffer size for all five scenarios to a limit of 100 data packets. Subsequently, the limit of data packets that a gateway can store was modified according to the number of IoT devices in each scenario. The optimal gateway buffer limit was deduced using the following formula:

(1) $$\mathrm{Bs}\;=\frac{N\times \mathrm{Ph}}{3600}\times \mathrm{Tb}$$

where

N—Number of IoT devices

Ph—data packets per device per hour

Tb—Desired buffer fill time

Using (1), we calculate the time required for the gateway to send IoT data packets to the blockchain network before the block is created. For example, in the case of a block time of 2 s, the gateway will send the data packets once every 1.8 s, thus considering the delays that may occur due to the blockchain network or the gateway. However, we see that in the case of networks where a block time of 30 s or 60 s is used, the Tb has been divided by 10. If a blockchain network with a block time of 30 s is used, the block fill time is 2.88 s instead of 28.8 s.

The reason for implementing this feature is because the gateway buffer would store far too many data packets, which would make sending them to the blockchain network very difficult, in most cases impossible. As we will see in the following, these changes in block time but also in the size of the gateway buffer affect the latency of the data packets, the size of the transactions, and the size of the block.

To be able to analyze all these performance metrics, test algorithms have been built and created by us using various programming languages, the main one being the Golang programming language. A more detailed description of the proposed blockchain IoT framework performance evaluation scenarios is presented below.

1. *Scenario 1:* In this scenario, we have simulated a Small-Scale Network with 5000 IoT devices. The data transmission frequency is once every 15 min with a total of four times per hour for each IoT device. The gateway buffer that encapsulates the data packets into transactions has been set to a limit of 10, 16, 30 and 100 data packets per transaction. The data packet throughput is calculated by dividing the total data packets to 3600 s, thus the data packet throughput for this scenario is 5.55 DPS. The total number of data packets in an interval of one hour for a number of 5000 IoT devices with a rate of once every 15 min transfer rate is 20,000.
2. *Scenario 2:* In the second scenario, we simulated a Medium-Scale Network with 10,000 IoT devices. In this case, we set a data transmission frequency of once every 15 min. Also, in this scenario, the gateway buffer is set to 20, 32, 60, and 100 data packets. The data transfer rate should be 11.11 DPS, and the total number of data packets transmitted in one hour according to the given specification is 40,000.
3. *Scenario 3:* For the third scenario, we simulated a Large-Scale Network with 20,000 IoT devices. Each device transmitted four data packets per hour thus resulting in a DPS of 22.22. In this scenario, the gateway buffer limit for the data packets is set to 40, 64, 120, and 100. The total number of data packets coming from a network with 20,000 IoT devices in an hour is 80,000.
4. *Scenario 4:* In this scenario, we emulated a Very Large-Scale Network with 500,000 IoT devices to observe the behavior of our framework on a larger number of IoT devices. The gateway buffer limit is set to 100, 1000, 1600, and 3000. Dividing the total data packets by 3600 s, we could obtain a total of 555.55 DPS. The total data packets transmitted in a one-hour interval would be 2,000,000.
5. *Scenario 5:* In the last scenario, we emulated a Massive Large-Scale Network with 1,000,000 IoT devices. In this scenario, we wanted to test the limits of the proposed framework by emulating a large number of IoT devices transmitting data packets at a 15 min interval. As in the other scenarios, the gateway buffer is set at 100, 2000, 3200, and 6000. The DPS obtained in this scenario would be 1111.11 if we divide the total data packets by 3600 s. The total data packets sent by 1 million devices in a one-hour interval at a frequency of four data packets once every 15 min would be 4,000,000.

### 6.2. Performance Metrics

In this section, we will discuss the performance metrics analyzed, and the results obtained. The performance metrics analyzed are data packet throughput, data packet latency, transaction size, block size, and data packet recovery time. These metrics provide a comprehensive understanding of the network’s efficiency and reliability. By evaluating these metrics, we can gain valuable insights into the strengths and weaknesses of the network’s performance.

#### 6.2.1. Data Packets Throughput Rate

**Figure 5:**
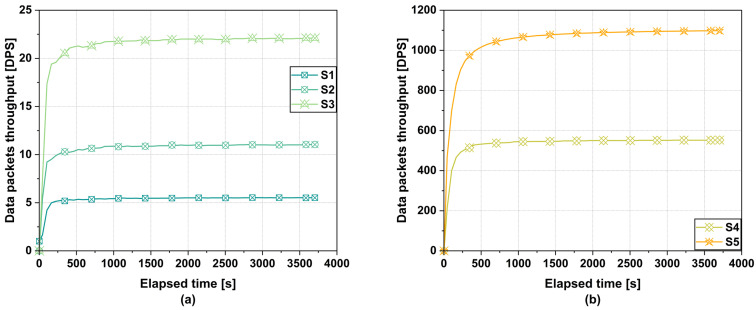
Data packets throughput for all 5 scenarios using a block time of 2 s, 30 s, and 60 s. (**a**) S1, S2, S3, (**b**) S4, S5.

The first performance metric measured is the data packet throughput rate (DPS), which is the rate of a blockchain network to process a specific number of data packets in a given period of time. According to the obtained results, which can be seen in Figure 5, the throughput differs from scenario to scenario as the number of emulated IoT devices and the interval at which they transmit data changes.

Regardless of the block time or the size of the data packet storage stack in the case of the gateway, there is no difference when it comes to data packet throughput. In the case of the first scenario for a network with a number of 5000 IoT devices, for all the three block times of 2 s, 30 s, 60 s, we obtained an average throughput of 5.28 DPS. The results are quite similar for the second case with a network of 10,000 IoT devices. Here, we obtained for all three block times an average throughput of 10.55 DPS. In the third scenario, we obtained an average throughput for the three block times of 21.09 DPS. For the fourth scenario, the results indicate a similar average throughput for all three block times of 527.65 DPS.

For the last scenario in a Massive-Scale Network with 1,000,000 IoT devices, we obtained an average throughput of 1035.16 DPS for all three block times. It should be specified that in the case of our evaluations, the TPS is to some extent replaced by the DPS. This is due to the way the data packets from IoT sensors are encapsulated and sent for verification and storage within our proposed blockchain IoT framework. Specifically, multiple data packets from different IoT sensors are encapsulated in a single transaction and then sent to the blockchain network for storage.

This approach of storing multiple data packets in a single transaction is much more efficient than storing a single data packet in an individual transaction. By batching packets of data, it optimizes the use of the blockchain network’s computing resources, reduces transaction overload, and minimizes network overheads, thus leading to faster processing time and reduced overall costs. On top of that, it facilitates and optimizes the scalability process, allowing the system to handle a larger volume of data packets without compromising its performance.

#### 6.2.2. Data Packets Latency

The next performance metric evaluated is data packet latency. This represents the elapsed time between the initiation and completion of a process, in this case being the time it takes for a transaction to be confirmed within the blockchain. For a transaction to be confirmed, it needs to add more blocks from the block where the transaction is located. In the case of our framework, we can consider a data packet to be valid if one or more blocks have been added from the block where the transaction is located.

**Figure 6:**
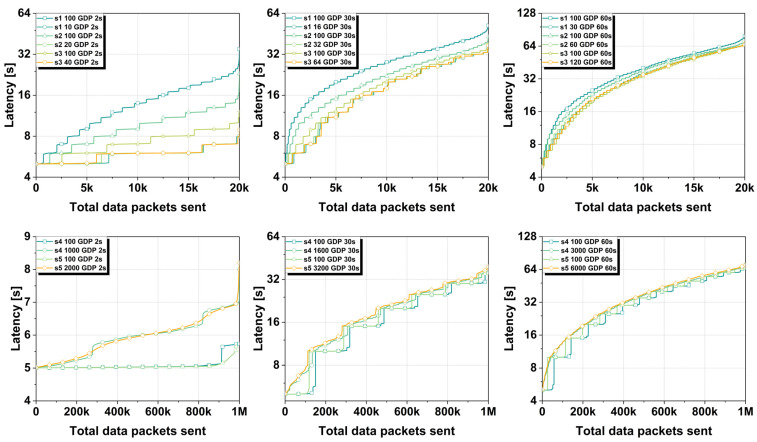
Latency results analyzed across all five proposed scenarios using block times of 2 s, 30 s, and 60 s. Each scenario was tested with both an optimized GDP and a standard 100 GDP configuration.

As with the other performance metrics, data packet latency was also evaluated for block time values of 2 s, 30 s, and 60 s with optimized gateway buffer size ranging from a limit of 10 to a limit of 6000 gateway data packets (GDP), for each of the five established scenarios. The results regarding the latencies of the data packets sent by IoT devices can be seen in Figure 6.

As can be seen, in the case of a block time of 30 s, for the first three scenarios, the average packet latency is not very different. When using a gateway buffer limit of 100 GDP for scenario 1, scenario 2, and scenario 3, we obtained average data packet latencies of 27.62 s, 22.46 s, and 20.04 s, respectively. For an optimized gateway buffer limit, we obtained lower latencies, ranging from 18.73 s for scenario 1, 18.01 s for scenario 2, and 18.01 s for scenario 3. For the last two scenarios, the average latencies are also quite close, ranging from 20.46 s for scenario 4 with a gateway buffer limit of 100 GDP, to 21.03 s for scenario 5 with an optimized gateway buffer limit of 3200 GDP.

In the case of using a block time of 60 s for the first three scenarios, we obtained latencies considerably closer to each other. The latencies vary from 35.25 s for a gateway buffer of 30 GDP for scenario 1 to 34.98 s in the case of a gateway buffer limit of 120 GDP for scenario 3. The same is true for the last two scenarios where a block time of 60 s was used. As can be seen, the latencies did not vary very much, ranging from 35.02 s to 38.05 s for a gateway buffer limit of 100 GDP and 6000 GDP, respectively. Significantly better results were achieved for networks with a 2 s block time. For the first three scenarios where a gateway buffer limit of 100 GDP was used, the lowest latencies were obtained. For scenario 1, we obtained a latency of 13.91 s, for scenario 2 a latency of 9.06 s, and for scenario 3 a latency of 7.02 s. In the case of an optimized gateway buffer limit, we obtained much better results, with much lower latencies compared to a 100 GDP limit. The average latency of the three scenarios in this case was 5.94 s.

For the last two scenarios, the results are slightly different. In the case of using an optimized gateway buffer limit, much higher latencies were obtained for the two scenarios than in the case of using a 100 GDP limit. For a 1000 GDP limit and a 2000 GDP limit, we obtained an average latency of 5.97 s for both. In the case of a 100 GDP limit, the average latency was 5.02 s.

The reason we obtained much lower latencies for an optimized gateway buffer limit in the first three scenarios compared to the last two scenarios is due to the way transactions are processed by the gateway. In the first three scenarios, the gateway buffer is filled much faster, which makes the data packets also be added faster to the blockchain network. In the last two scenarios, even though we have a large number of IoT devices, the gateway has to wait for the buffer limit to be reached in order to send the data packets for processing and storage in the blockchain network. In the last two scenarios where we have a network with numerous IoT devices, the gateway’s buffer limit is 100 GDP, an ideal point where latency is compromised with the storage efficiency of data packets in transactions but also in the blockchain network.

#### 6.2.3. Transaction Size

**Figure 7:**
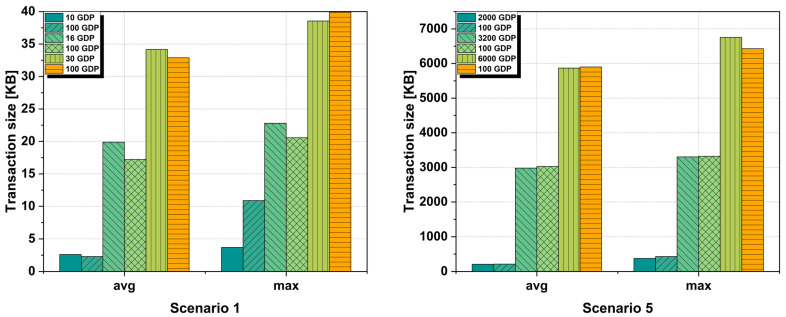
Transaction size comparison for scenario 1 and scenario 5 using an optimized GDP and a standard 100 GDP configuration.

Another performance metric analyzed is the transaction size within our proposed blockchain IoT network. As specified in Section 4, in our framework, multiple data packets are encapsulated, according to each scenario, in a single transaction. This can lead to an increase in the transaction size from values of a few bytes in the case of a single transaction, to values of tens of megabytes for transactions having data packets in the order of tens of thousands. The results can be seen in Figure 7 where we chose to plot scenario 1 and scenario 5 for the two networks with the smallest and largest number of IoT devices.

Some important characteristics that have an impact on the transaction size, regardless of the scenario analyzed, are the block time and the gateway buffer limit chosen. To exemplify this, we analyze the transaction sizes for scenario 1 with a total of 5000 IoT devices and scenario 5 where we have a total of 1,000,000 emulated IoT devices that generate and transmit data packets to the gateway four times in a one-hour interval.

In the case of a blockchain network with a block time of 2 s and a GDP of 100, the average size of a transaction is 2.60 KB with a maximum size of 3.68 KB. For an optimized GDP of 10, the average size is 2.29 KB and a maximum size of 3.68 kB. For a block time of 30 s for a GDP of 100, the average transaction size is 15.83 KB with a maximum transaction size of 19.13 KB. For the same block time but with an optimized GDP of 16, the average transaction size is 17.55 KB with a maximum transaction size of 20.29 KB. For a block time of 60 s with a GDP of 100, the transactions had an average transaction size of 32.88 KB with a maximum transaction size of 39.90 KB. In the case of an optimized GDP of 30, the average transaction size is 34.16 KB with a maximum transaction size of 38.54 KB. For scenario 5 in the case of a blockchain network with a block time of 2 s and a GDP of 100, the average transaction size is 211.45 KB with a maximum transaction size of 430.48 KB. The average transaction size for an optimized GDP of 200 is 207.38 KB with a maximum transaction size of 375.38 KB. In the case of using a network with a block time of 30 s and a GDP of 100, the transactions had an average transaction size of 3027.48 KB with a maximum transaction size of 3319.54 KB. For the same 30 s block time with an optimized GDP of 3200, the transactions had an average size of 2976.18 KB and a maximum size of 3302.10 KB. For the same scenario, but with a block time of 60 s and a GDP of 100, the transactions had an average size of 5900 KB and a maximum size of 6428.62 KB. For an optimized GDP of 6000 for the same block time, the average transaction size is 5868.06 KB with a maximum transaction size of 6752.29 KB.

#### 6.2.4. Block Size

**Figure 8:**
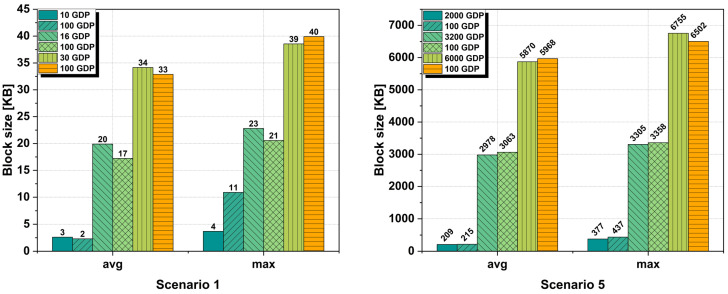
Average block size for scenario 1 and scenario 5 with a block time of 2 s, 30 s, and 60 s using an optimized GDP and a standard 100 GDP configuration.

Another analysis was done on the size of a block within the blockchain network. As with transactions, the block size is also influenced by the block time and the gateway buffer limit chosen. The size of the block is automatically influenced by the number of transactions to be added but also by their size. The more transactions a block has, the larger its size will be. The block size results can be seen in Figure 8. And in this case, as in the case of analyzing the average and maximum size of a transaction, we considered scenario 1 where a network with 5000 IoT devices is deployed and scenario 5 with a network where a total of 1,000,000 IoT devices were deployed.

The analysis was performed for all three block times of 2 s, 30 s, and 60 s where a standard GDP of 100 and a GDP optimized to each scenario was chosen. Following the analysis, the results show that indeed, both the block time and the gateway buffer size have a major influence on the size of a block. However, whether we choose a standard GDP of 100 or an optimized GDP, the differences between the two cases are not very large. For example, in the case of scenario 5 for a block time of 60 s with a GDP of 100, we have an average block size of 5967.92 KB with a maximum block size of 6502 KB, while in the case of an optimized GDP of 6000 and the same block time, we have an average block size of 5870.42 KB with a maximum block size of 6754.82 KB.

As can be seen in Figure 8, in both scenario 1 and scenario 5, the smallest block sizes were obtained for a block time of 2 s even if we used a standard or an optimized GDP. The smaller the block time, the smaller the block size, because it has fewer transactions which in turn encapsulates fewer data packets coming from IoT devices.

Following this analysis of the block size and transaction size, it is noticeable that for each blockchain network, a suitable block time is needed so that the blocks do not remain empty in the case of a network with 5000 IoT devices, or that their size is too large in the case of a network with over 1 million IoT devices.

These two issues mentioned above can lead to various problems. A block time of 2 s for an IoT device network of 5000 IoT devices can lead to a very large number of blocks devoid of transactions which could be a problem for the packet search algorithm.

In the case of the second problem, a much larger block size can lead to problems in efficiently distributing the blocks throughout the blockchain network. So, a good solution is the one presented by us after the analysis where a block time and a GDP must be chosen corresponding to each network, be it a network of 5000 IoT devices or 1,000,000 IoT devices.

#### 6.2.5. Data Packets Retrieval Time

**Figure 9:**
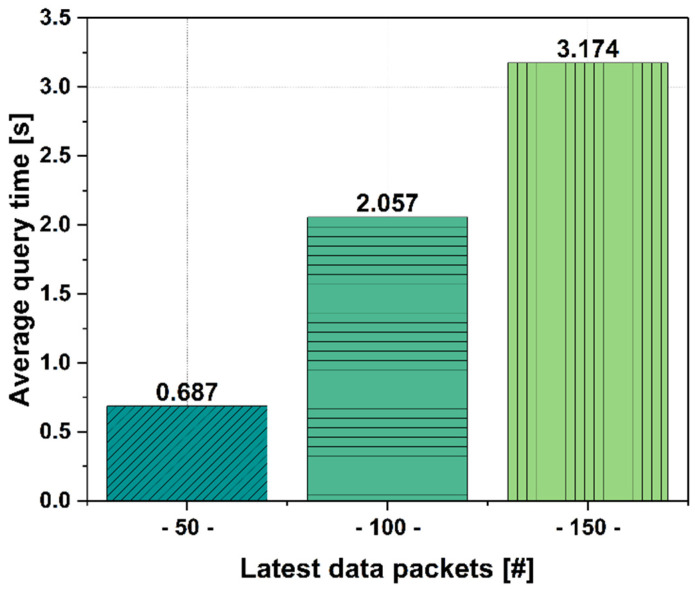
Average query time for the most recent 50, 100, and 150 data packets.

Another analysis was performed on the retrieval time of data packets in the blockchain network. The analysis was performed to test the recovery of the last 50, 100, and 150 data packets. The results obtained are shown in Figure 9. 

In each case, we ran the algorithm 10 times to be able to calculate the average query time. As can be seen, the results show that our data retrieval solution using the proposed binary search algorithm implemented in this framework is very efficient even for retrieving a total of 150 data packets.

#### 6.2.6. Storage Impact of High-Density IoT Data

An important consideration for the long-term viability of any blockchain-based IoT framework is the rate at which the blockchain ledger grows as a result of continuous data transmission. In this framework, each IoT data packet stored on-chain has an estimated size of approximately 300 bytes. Based on this assumption, a series of calculations was conducted to assess the blockchain ledger size over daily, monthly, and yearly intervals for the five experimental scenarios defined in Table 1. In Scenario 1, which includes 5000 IoT devices transmitting data every 15 min, the blockchain ledger is expected to grow by roughly 0.13 GB per day, resulting in an annual increase of approximately 46.52 GB. Scenario 2, with 10,000 devices under the same conditions, produces about 0.27 GB of new data daily, or 97.89 GB per year. Scenario 3, involving 20,000 devices, yields approximately 0.54 GB per day and 195.78 GB annually. For larger deployments, such as Scenario 4 with 500,000 devices, the ledger growth becomes more substantial, reaching around 13.41 GB per day and nearly 4.89 TB over the course of a year. In Scenario 5, where 1,000,000 devices are active, the projected ledger expansion reaches 26.82 GB per day, corresponding to approximately 804.66 GB per month or 9.79 TB per year.

This cumulative storage footprint is primarily influenced by the number of data packets transmitted and their individual sizes, rather than by how they are grouped into transactions. Whether data packets are submitted to the blockchain individually or aggregated into larger transactions by the gateway nodes, the total volume of data written to the ledger remains largely the same. Nonetheless, transaction batching plays an important role in reducing network and consensus overhead, improving processing efficiency, and optimizing block utilization. It is also important to note that block time does not significantly affect the total ledger size. While shorter block intervals result in a greater number of smaller blocks and longer intervals produce fewer, larger blocks, the overall volume of data stored on-chain is dictated by packet frequency and size. However, block time can influence other performance aspects of the blockchain network, such as consensus traffic, block propagation delays, and real-time responsiveness.

To facilitate a clear and comprehensive comparison of our experimental results, Table 3 consolidates the key performance metrics, including data packet throughput (DPS), latency, block size, transaction size, and average query time across the different deployment scenarios.

## 7. Discussion

In this section, we discuss some aspects related to security, scalability, and decentralization of the proposed new framework. These previously stated elements, security, scalability, and decentralization are key features underlying the technology known as blockchain. However, in specialized studies, it has been concluded that it is quite difficult to obtain all these three criteria into the blockchain network. This aspect is known as blockchain trilemma [43], and it refers to the problem faced by any blockchain network. In the following, we will see that our framework also encounters this problem, where it was necessary to drop (or reduce the importance of) one of the three characteristics, namely decentralization. The reason why we decided to give up decentralization to some extent is due to the blockchain application that we used to create our framework, namely the Hyperledger Besu application. This application is designed to provide a high level of security through consensus protocols such as QBFT and IBFT 2.0. Moreover, the entities in charge of verifying transactions and adding blocks within a network must be authorized and are put in that position by the other entities. This leads to meticulous access control within the blockchain network.

The third feature, scalability, is also present in our framework and can be seen by the results obtained by benchmarking the five scenarios. We have managed by various methods to obtain a relatively high throughput of 1035.16 DPS for a network with 1 million IoT devices which confers confidence that our framework can be scalable. By carefully modifying the network, the nodes, and by introducing other elements such as a load balancer, this framework could be scaled up to a much higher DPS than the one obtained in this work.

By giving up one of the three features of our framework, namely decentralization, we managed to maintain scalability and a high level of security which makes our system suitable for IoT networks and systems where we can find a multitude of devices transmitting many data packets over a long period of time.

Following the evaluations of the proposed scenarios, we determined that for an IoT network with 1 million active devices transmitting every 15 min, a blockchain with a 2 s block time is recommended. In this configuration, a block size of 200 KB is ideal for faster block distribution across the network and quicker acknowledgment of data packets. However, in scenarios with a 60 s block time and a buffer size of 3200 or 6000 data packets, we observed higher data packet latencies. As the buffer size increases, the resulting block size grows larger, making it more difficult to propagate efficiently through the blockchain network. This can lead to delays in block distribution and slower acknowledgment of data packets, ultimately impacting the performance and responsiveness of the network.

For a smaller network with 5000 IoT devices, the data show that a 60 s block time is more appropriate than 2 s block time. A 60 s block time allows more transactions to be grouped into a block, resulting in a higher number of data packets per block. On the other hand, a 2 s block time would not provide enough time for the gateway to fill transactions, leading to fewer data packets per block and the potential creation of empty blocks.

In Table 4, we present a comparative analysis of six recent blockchain-based IoT frameworks alongside our proposed solution. The comparison focuses on measurable technical attributes such as maximum number of simulated devices, transaction throughput, latency, block time, real-time data retrieval capabilities, consensus mechanisms, and implementation status. This structured evaluation highlights the extent to which each approach addresses scalability, performance, and practical applicability in large-scale, high-density IoT environments.

While the proposed solution offers significant improvements through the use of blockchain technology in IoT systems, it also has some limitations. The first of these relates to the total number of IoT devices that the proposed framework can manage. In our solution, we were able to manage up to one million devices. However, managing more than one million devices can lead to problems such as reduced system performance, increased response time, and increased complexity in data management. Another limitation of the solution is the blockchain gateway’s grouped management of IoT data in a buffer. This approach works best when the data packet size is between 25 and 50 bytes. In our tests, the packet size was between 20 and 25 bytes, which was within the set limits, but larger data sizes could impact performance.

Several solutions can be implemented to overcome these limitations. For example, the use of advanced scaling strategies, such as fragmentation of the blockchain network, could enable load balancing across multiple nodes, making it easier to manage a larger number of devices without compromising performance. At the same time, optimizing data management algorithms can reduce response time and simplify the management of a larger number of devices. Another useful measure would be to adjust the size of data packets to better match system requirements, improving processing efficiency and avoiding potential bottlenecks.

## 8. Conclusions

In this paper, we presented a blockchain-based framework designed for large-scale IoT systems that require secure, efficient, and scalable data management. The framework was developed and tested in the USV cloud platform [44] and integrates several core components, including IoT sensors, gateway nodes, a permissioned blockchain network using the QBFT consensus mechanism, smart contracts, retrieval nodes, and a web-based dashboard for real-time data visualization. The gateway nodes aggregate and buffer data packets from IoT devices, then encapsulate and sign them before submitting transactions to the blockchain network. This proposed batching mechanism reduces the total number of transactions, significantly improving blockchain efficiency, especially in dense IoT deployments. Smart contracts ensure that data are securely stored and retrieved according to pre-defined rules, while a binary search-based retrieval algorithm accelerates access to current data. A conventional database complements the blockchain ledger by supporting fast queries and device–user mapping for dashboard presentation.

The framework was experimentally evaluated across five scenarios, ranging from 5000 to 1,000,000 IoT devices. Results demonstrated the system’s ability to sustain data packet throughput from 5.28 DPS to 1035.16 DPS, depending on the network size and configuration. Additionally, data retrieval latency remained consistently low—under 3.2 milliseconds—even in the largest deployment. These results confirm the high performance and scalability of the proposed framework. Security is ensured through the QBFT consensus protocol, which provides Byzantine fault tolerance without the computational overhead of PoW, allowing fast block times without compromising trust or integrity. The novel framework supports multiple communication protocols and can be adapted to both small- and large-scale deployments, making it highly versatile. The framework developed is specifically designed to address the scalability, security, and efficiency challenges of large-scale IoT networks, and is made freely available to the academic community in an open-access manner on GitHub [45], providing researchers and practitioners with a robust toolset to explore, modify, and contribute to its ongoing development and enhancement.

In conclusion, the proposed framework demonstrates a viable solution for managing massive volumes of IoT data with blockchain technology, combining the immutability and traceability of distributed ledgers with the speed and flexibility of off-chain components. Its performance and adaptability make it suitable for a broad range of IoT applications, from smart cities to industrial monitoring.

## Figures and Tables

**Table 1:** Configuration options for each blockchain node from the proposed framework.

Option	Value
tx-pool-limit-by-account-percentage	*0.5*
tx-pool	*sequenced*
cache-last-blocks	*32*
tx-pool-max-size	*8192*
data-storage-format	*BONSAI*
p2p-port	*port-number*
bootnodes	*bootnode or list of bootnodes*
rpc-http-host	*0.0.0.0*
rpc-http-enabled	*true*
rpc-http-api	*ETH, NET, QBFT*
host-allowlist	*list of hosts*
rpc-http-cors-origins	*all*
rpc-http-port	*random port*
min-gas-price	*0*
rpc-ws-enabled	*true*

**Table 2:** Established scenarios for the testing of the proposed blockchain IoT framework.

Scenario	Device Count [#]	Data Transmission Frequency	Gateway Buffer Size [#]	Block Time Options	Expected Throughput [DPS]	Estimated Total Data Packets [#]
*Scenario 1*	5000	Every 15 min	10, 16, 30, 100	2 s, 30 s, 60 s	5.55	20,000
*Scenario 2*	10,000	Every 15 min	20, 32, 60, 100	2 s, 30 s, 60 s	11.11	40,000
*Scenario 3*	20,000	Every 15 min	40, 64, 120, 100	2 s, 30 s, 60 s	22.22	80,000
*Scenario 4*	500,000	Every 15 min	100, 1000, 1600, 3000	2 s, 30 s, 60 s	555.55	2,000,000
*Scenario 5*	1,000,000	Every 15 min	100, 2000, 3200, 6000	2 s, 30 s, 60 s	1111.11	4,000,000

**Table 3:** Summary of performance metrics for the proposed blockchain IoT framework.

Performance Metrics		Data Transfer Rate [DPS]	Latency		Block Size [KB]	Transaction Size [KB]	Average Query Time [s]		
			100 GDP	Opt. GDP			50	100	150
Scenario 1	2 s	5.28	13.91	5.94	2 KB	1.5 KB	0.68	2.05	3.17
	30 s	5.28	27.62	18.73	19 KB	16 KB	0.68	2.05	3.17
	60 s	5.28	34.98	35.25	35 KB	32.5 KB	0.68	2.05	3.17
Scenario 2	2 s	11.11	9.03	5.94	3 KB	2.07 KB	0.68	2.05	3.17
	30 s	11.11	22.46	18.01	33 KB	31.91 KB	0.68	2.05	3.17
	60 s	11.11	34.98	34.98	64 KB	63.98 KB	0.68	2.05	3.17
Scenario 3	2 s	21.09	7.02	5.95	5 KB	3.94 KB	0.68	2.05	3.17
	30 s	21.09	20.04	18.01	63 KB	60.91 KB	0.68	2.05	3.17
	60 s	21.09	35.02	34.98	125 KB	125.86 KB	0.68	2.05	3.17
Scenario 4	2 s	527.65	5.97	5.97	90 KB	93.94 KB	0.68	2.05	3.17
	30 s	527.65	20.46	21.03	1.47 MB	1.50 MB	0.68	2.05	3.17
	60 s	527.65	35.02	38.05	3.03 MB	3.09 MB	0.68	2.05	3.17
Scenario 5	2 s	1035.16	5.02	5.97	0.2 MB	187 KB	0.68	2.05	3.17
	30 s	1035.16	21.03	20.1	2.9 MB	3 MB	0.68	2.05	3.17
	60 s	1035.16	35.02	38.5	6.7 MB	6.18 MB	0.68	2.05	3.17

**Table 4:** Comparative analysis of blockchain-based IoT frameworks across key performance metrics.

Reference	Max IoT Devices	Throughput [TPS/DPS]	Latency [s]	Block Time [s]	Data Retrieval Support	Consensus Type	Practical Implementation
[23]	N/A	N/A	N/A	12 s	✗	PoW	✗
[24]	~300	800 TPS	4.91 s	N/A	Partial	PoA	✓
[25]	N/A	N/A	N/A	N/A	Partial	PoA	✗
[26]	N/A	N/A	N/A	2–10 s	✗	LCA	✗
[27]	N/A	N/A	N/A	N/A	✗	LCA	✗
[28]	N/A	~30–40 TPS	~7–9 s	5–15 s	Limited	Shard-local	✗
Our approach	1,000,000	1035.16 DPS	5–6 s	2/30/60 s	✓	QBFT	✓

## Data Availability

Dataset available on request from the authors. The raw data supporting the conclusions of this article will be made available by the authors on request. The developed framework is available in an open-access manner on GitHub reference [23] of the paper.
